# Message in a Bottle: Upgrading Cardiac Repair into Rejuvenation

**DOI:** 10.3390/cells9030724

**Published:** 2020-03-15

**Authors:** Carolina Balbi, Ambra Costa, Lucio Barile, Sveva Bollini

**Affiliations:** 1 Laboratory of Cellular and Molecular Cardiology, Cardiocentro Ticino Foundation, 6900 Lugano, Switzerland; carolina.balbi@cardiocentro.org; 2 Regenerative Medicine Laboratory, Dept. of Experimental Medicine (DIMES), University of Genova, 16132 Genova, Italy; costa.ambra93@gmail.com; 3 Laboratory for Cardiovascular Theranostics, Cardiocentro Ticino Foundation, 6900 Lugano, Switzerland; 4 Faculty of Biomedical Sciences, Università della Svizzera Italiana, 6900 Lugano, Switzerland

**Keywords:** paracrine effect, extracellular vesicles, exosomes, cardiac repair, angiogenesis, myocardial renewal, regeneration

## Abstract

Ischaemic cardiac disease is associated with a loss of cardiomyocytes and an intrinsic lack of myocardial renewal. Recent work has shown that the heart retains limited cardiomyocyte proliferation, which remains inefficient when facing pathological conditions. While broadly active in the neonatal mammalian heart, this mechanism becomes quiescent soon after birth, suggesting loss of regenerative potential with maturation into adulthood. A key question is whether this temporary regenerative window can be enhanced via appropriate stimulation and further extended. Recently the search for novel therapeutic approaches for heart disease has centred on stem cell biology. The “paracrine effect” has been proposed as a promising strategy to boost endogenous reparative and regenerative mechanisms from within the cardiac tissue by exploiting the modulatory potential of soluble stem cell-secreted factors. As such, growing interest has been specifically addressed towards stem/progenitor cell-secreted extracellular vesicles (EVs), which can be easily isolated in vitro from cell-conditioned medium. This review will provide a comprehensive overview of the current paradigm on cardiac repair and regeneration, with a specific focus on the role and mechanism(s) of paracrine action of EVs from cardiac stromal progenitors as compared to exogenous stem cells in order to discuss the optimal choice for future therapy. In addition, the challenges to overcoming translational EV biology from bench to bedside for future cardiac regenerative medicine will be discussed.

## 1. Introduction: Cardioprotection Versus Regeneration: Where do We Stand?

### 1.1. Cardioprotective Mechanisms: Current Limits and Future Perspective 

Contrary to lower vertebrates, the adult mammalian heart cannot withstand prolonged injury, such as severe ischaemia, being endowed with a defective repair programme as the one and only emergency and life-saving mechanism. Indeed, evidence dating back to 1977 has shown a time-dependency spread of the infarction wavefront during ongoing ischaemia [1]. It is well known now that irreversible ischaemic myocardial cell injury develops in an increasing number of compromised cells as the duration of coronary occlusion is prolonged up to 2 to 3 h [1]. In response to ischaemia, the intrinsic cardiac repair mechanism within the adult heart represents an impaired wound healing response, leading over time to cardiomyocyte (CM) hypertrophy with the spreading of fibrosis, which can result in the detrimental remodelling of the cardiac chambers and heart failure. Currently, the best cure for cardiac dysfunction and heart failure is represented by heart transplantation, which is heavily influenced by donor supply and compatibility.

Several cardioprotective strategies have consistently been utilised with the aim of protecting the heart during periods of ischaemia or hypoxia followed by reperfusion. Hypothermia (32–35 °C) activates survival signalling mechanisms that involves either the extracellular signal-regulated kinases (ERK1/2) and/or the Akt/phosphoinositide 3-kinase/mammalian target of rapamycin pathways [2] leading to a reduction of myocardial infarct size in rabbits [3], sheep [4], pigs [5] and rats [6]. Conditioning phenomena [7] are also known to exert cardioprotective effects. This process includes three types of manipulations: (1) ischaemic preconditioning (IPC), with cycles of occlusion (5 min) and reperfusion (5 min) before sustained coronary occlusion [8]; (2) ischaemic postconditioning (POC) for which cycles of occlusion and reperfusion are applied at the onset of reperfusion after acute myocardial infarction (AMI) [9]; and finally the remote ischaemic preconditioning (RIPC) by which the conditioning is performed remotely by inflating a blood pressure cuff placed on the upper limb [10]. Several potential signalling and molecular pathways have been proposed as intracellular mechanisms for cardioprotection in conditioning phenomena. Heusch et al. have recently formulated the hypothesis that most of these processes are affecting mitochondrial function and converge on this organelle as the final effector [8]. Mitochondrial damage negatively affects CMs function as it leads to disruption of oxidative phosphorylation, Ca^(2+)^ dyshomeostasis and increased oxidative stress. The process is initiated when the high-conductance channel located at the contact sites between the inner and outer mitochondrial membranes, the so-called Mitochondrial Permeability Transition Pore (MPTP), open for longer terms and dissipates the inner mitochondrial membrane potential. The activation of PI3K, phosphoinositide-dependent kinase, Akt and ERK (RISK Pathway) [11,12] by IPC [13] and POC [14] targets the glycogen synthase kinase 3β (GSK 3β) and inhibit the opening of MPTP [7]. NO is an essential molecule in IPC, POC and RIC, besides the well-known mechanism implying the increase cGMP formation and activation of PKG [15]. NO targets mitochondrial proteins through the nitrosination or nitrosylation process [16,17]. Nitrosation of mitochondrial respiratory chain complex I [18] and mitochondrial connexin 43 (mtCx43) [19] are crucial in the cardioprotective effects mediated by IPC and RIPC, respectively. IPC induces also translocation of mtCx43 into the mitochondria through a heat shock protein-dependent transport mechanism [7,20], which determines bioenergetics stabilisation of mitochondria by increasing potassium uptake [19,20]. The ATP-dependent potassium channel (K_ATP_) at inner mitochondrial membrane [21,22] is involved in the protective influence exerted by IPC [23], POC [24] and RIPC [25]. Although mechanisms are not completely clear, the mitochondrial K_ATP_ channel upon its activation (following mitochondrial translocation of PKC) releases reactive oxygen species (ROS) which, in turn, activates the PKCε in a positive feedback loop [7]. There is additional evidence showing anaesthetic agents such as opioids enhance RIPC-induced cardioprotection in infarcted patients [26] which is in agreement with observations that the beneficial effect of RIPC is inhibited by the opioid receptor blocker naloxone [27], although mechanisms that govern this effect have not been completely elucidated.

Pharmacological intervention can reduce morbidity and mortality associated with myocardial infarction [28]. Current international guidelines support the cardioprotective benefits of beta-blockers in perioperative myocardial infarction as they contribute to reducing the force of contraction and heart rate, thus leading to decreased myocardial oxygen demand [29,30]. Beta-blockers also suppress monocyte activation and inflammatory cytokine response [31]. The latter mechanism has been also postulated as the basis of cardioprotective effects of beta-blockers when orally administered in rats in chronic settings, 12 weeks after large myocardial infarction, where attenuation of TNF-α and IL-1β expression was observed [32]. 

Recently, cell therapy has been studied with the goal of direct “cell replacement” of the injured myocardium with newly formed functional cardiomyocytes, especially via transplantation of pre-committed or undifferentiated stem cells. There was initial excitement over the adult cell transplantation approach for its relative ease of use and good safety profile, but the initial mechanistic hypothesis has been lately questioned. To date, cell therapy has been effective in reducing cardiomyocyte apoptosis and tissue fibrosis, while promoting angiogenesis and immunomodulation of inflammation, likely via modulatory stimulation of the local microenvironment [33,34]. This is leading to a paradigm shift, where the trophic molecules secreted by the transplanted cells are considered more critical than the differentiation potential of the cells. Indeed, modulation of paracrine cardiac endogenous mechanisms that exploit stem/progenitor cell secretory capacity has become an appealing strategy for future cardiovascular medicine. In this scenario, cell-secreted factors could be envisioned to enhance and optimise defective cardiac repair programme, by means of supporting the survival of resident cardiomyocytes, activating therapeutic angiogenesis and modulation of inflammation, in order counteract excessive fibrosis. 

### 1.2. Gap between Experimental Animal Research and Clinical Outcome Studies

Cardioprotective approaches often remain confined at preclinical levels. Experimental settings in small animals mainly aim at unveiling new specific molecular mechanisms by pointing out single aspects. Complex systems, including risk factors, comorbidities and co-medications, are intentionally avoided [35,36]. Although large animals are more suitable for setting up studies that take confounders into consideration, as they most resemble human physiological and pathophysiological processes [37], robust data from large preclinical models of myocardial ischaemia/reperfusion are still missing [36]. Ethical concerns, complex study designs including long time follow-up, proper housing and breeding conditions, that inevitably increase costs are some of the aspects that limit the use of large animals in preclinical research [37]. Even when cardioprotective agents have been evaluated in large animals using rigorous experimental conditions, based on principles of clinical trials (investigator blinding, randomisation, exclusion criteria) [38,39], the scientific community has faced substantial failure in translation.

The most daring challenge for newly studied approaches is to gain cardioprotective effects over reperfusion. Early reperfusion per se may be sufficient to salvage myocardium at risk [36]. Adjunct cardioprotection that rescues reperfused myocardium can be achieved in a very limited time window [36,40]. In patients after acute myocardial infarction (MI), intracoronary or systemic delivery of the cardioprotective agent, soon after the percutaneous coronary angioplasty, is a desirable option, as it has been implemented with systemic thrombolysis [41]. This approach has never been comprehensively tested to evaluate the cardioprotective effects of stem/progenitor cells within the heart regeneration scenario [40]. Indeed, studies in small animals have investigated cells in a setting of permanent coronary ligation [42], and several others have targeted chronically scarred myocardium [43,44,45]. Clinical trials based on the injection of cells in autologous settings suffer from intrinsic delay as time-consuming tissue harvesting and cell processing are required first. As for the conditioning protocols, they have been tested in humans, with some limitations. For example, IPC can be used in elective settings such as percutaneous transluminal coronary angioplasty (PTCA), and it can be applied before reperfusion to patients undergoing a coronary artery bypass graft (CABG). POC can only be applied at the immediate onset of reperfusion. RIPC would be the most clinically relevant methodology as it is applied before and during evolving infarction; however, two recently published studies showed no benefit of RIPC on clinical outcomes following cardiac surgery [46,47].

In this context, cell-secreted factors and/or vesicles might represent an allogeneic and off-the-shelf donor-derived product to be tested in the acute reperfusion phase. However, prior to retracing the road of canonical stem cell therapy and receiving similar dispiriting results, multi-centred rigorous preclinical tests in large animals are absolutely needed in close analogy to clinical trials [36]. 

### 1.3. Cardiac Regeneration by Rejuvenation: Challenging the Postnatal Memory Loss

In the last few years, there have been high expectations that endogenous stromal cardiac mesenchymal cells, originally defined as cardiac progenitor cells (CPCs), were endowed with cardiomyogenic and cardiovascular commitment potential. Indeed, CPCs have been long considered as an appealing source for cardiac regeneration via their in situ reactivation, expansion and differentiation or, alternatively, by means of trans-differentiation of either autologous or allogeneic ones transplanted into the injured myocardium [48,49,50,51,52,53,54,55] Multiple independent investigators have reported several heterogeneous CPC populations according to specific isolation protocols and marker expression patterns [54,55,56,57,58,59,60]. Notably, CPCs and, in particular, epicardium-derived progenitor cells, namely EPDCs, have been suggested to play a pivotal role in modulating the underlying myocardium and directly supporting to coronary vascular smooth muscle cells, cardiac fibroblasts and possibly a small proportion of ventricular cardiomyocytes. While broadly active during embryonic development, EPDCs become almost completely quiescent soon after birth, being unresponsive to cardiac injury and with significant loss of developmental memory [50,61,62,63]. Therefore, insights from endogenous mechanisms driving EPDC activation and instructing their role in organ formation during cardiogenesis could be exploited to reinstate their embryonic potential in adulthood, thus enhancing their therapeutic relevance. This has been the case for murine WT1-positive EPDCs primed with the cardio-active peptide thymosin β4, as this signalling cue restored their cardiomyogenic and cardiovascular potential following myocardial infarct, albeit with quite limited efficiency [64].

More recently, a growing body of evidence has questioned the true cardiomyogenic potential of adult CPCs within the diseased myocardium, with much scepticism and concern on the contribution of non-cardiomyocyte cells to post-injury de novo cardiomyogenesis. Indeed, several studies based on accurate CPC genetic lineage tracing have debated their trans-differentiation capacity into functional adult cardiomyocytes [65,66,67,68,69]. Nevertheless, a much less controversial paradigm suggests that, while their cardiovascular differentiation potential may not be therapeutically relevant, their endogenous reactivation might ameliorate and preserve cardiac function during disease progression or following injury. This may be mainly achieved via paracrine modulatory effects on the neighbouring resident cardiac cells, as confirmed both via injection of the CPC-conditioned medium containing all the cell-secreted soluble factors, or by their in situ stimulation [70]. In light of such evidence, endogenous CPCs may still represent an appealing cell source for cardiac repair and regeneration by means of ad hoc reactivation along with the recapitulation of their pro-active secretory paracrine potential.

A major challenge for cardiac regeneration is the loss of functional muscle tissue due to cell death or premature senescence of mature contractile cardiomyocytes during disease or severe injury (i.e., myocardial infarction) [71]. While pharmacological treatment and interventional cardiology can significantly contribute to preserving compromised cardiomyocytes after acute and limited-in-time ischaemia, there is still a worldwide unmet clinical urge for myocardial renewal, with structural and functional bona fide reconstitution of the lost cardiomyocytes. Indeed, the injured mammalian heart can only cope by means of a meagre repair programme, rather than via true regeneration, as comprehensively reviewed in Vujic et al. [72]. Given that stem/progenitor cell cardiomyogenic trans-differentiation has been shown to be not that therapeutically relevant, an alternative strategy is to trigger resident cardiomyocyte proliferation. The foetal myocardial tissue harbours remarkable renewal activity via cardiomyocyte hyperplasia. Nevertheless, this potential severely declines over time to almost complete unresponsiveness, with very modest turn-over maintenance of pre-existing cardiomyocytes during adulthood (about 1% per year in the mature human heart) [73,74,75]. Despite clearly insufficient to offset the loss of billions of cardiomyocytes following myocardial infarct, a key question is whether this rate of proliferation can be therapeutically enhanced. Notably, it has been shown that in the neonatal mouse heart, full regeneration following injury can be underpinned by the active proliferation of existing mononuclear cardiomyocytes. Unfortunately, this mechanism is transient, being lost after the first week of birth (P7), with the transition from complete regeneration to scarring/fibrosis [76].

Hence, the adult cardiac tissue experiences a sort of “memory loss” affecting specific embryonic/early postnatal mechanisms that drive regenerative effects to the detriment of defective repair. This regenerative capacity needs to be “rejuvenated” by specific stimuli, in order to restore the regenerative potential in the damaged adult heart, by addressing CPC restoration and resident cardiomyocyte bona fide proliferation. Indeed, accumulating evidence from state-of-the-art preclinical models in rodents and lower vertebrates, like the zebrafish, suggest that signals activating cardiomyocyte proliferation elicit global activation within the myocardium, pointing at paracrine mechanisms as master drivers of endogenous tissue reactivation [76,77]. The relevance of such intercellular communication has been further confirmed by recent studies emphasising the instructing role of macrophages in driving angiogenesis during murine neonatal heart regeneration [78] and of regulatory T cells in promoting foetal and maternal cardiomyocyte proliferation during pregnancy and after myocardial infarction [79]. 

### 1.4. Moving Forward Towards Paracrine Therapy

Considering all this evidence, paracrine stimulation of cardiac tissue might represent an ideal approach to “reboot” the endogenous potential of the adult heart, so as to recapitulate the neonatal reparative and regenerative profile. To further pursue such paracrine dogma for future cardiovascular therapy, the search is now on to identify the ideal stem cell source with the optimal cardio-active secretome (the whole of soluble paracrine molecules secreted by the cell), in order to turn back the myocardial rejuvenation clock and activate the right paracrine instructions within the injured myocardium. In this perspective, the secretome of different stem and progenitor cells have demonstrated the enhancement of cardiac repair mechanisms by providing cardioprotection, increased angiogenesis and modulation of scarring, while counteracting the worsening of cardiac function. These cells have also been described to target endogenous CPCs to stimulate resident pre-existing cardiomyocyte to re-enter the cell cycle [80,81,82,83,84,85,86]. In this scenario, interest has been growing towards a strategic role for progenitor cell-secreted extracellular vesicles (EVs) as immunologically inert vehicles for regenerative activity [87,88]. EVs are a heterogeneous population of nano/micro-scaled lipid vesicles with potent paracrine potential, including exosomes (Exo) and microvesicles (MV), and are increasingly being used in preclinical research for cancer and cardiac disease [89,90,91]. 

In this review, we will provide a comprehensive overview on the paracrine potential of different stem cells, with specific attention on the key role of their secreted EVs, in enhancing cardiac repair up to regeneration. In particular, we will compare the biological relevance of endogenous CPCs versus other stem/progenitor cells to discuss the optimal candidate source to be exploited for future therapy.

## 2. EV Biology in the Paracrine Era

### 2.1. Extracellular Vesicles: One Name, Many Faces

The field of EV biology is becoming extremely appealing in both preclinical and clinical research, as shown by the increasing number of relevant publications. Indeed, as EVs are critical modulators in physiological and pathological conditions, their impact on tissue function and role within inter-cellular paracrine communication may be pivotal, thus representing an interesting and novel therapeutic and/or diagnostic tool [92]. EVs are enriched with different bioactive proteins, metabolites, biolipids and genetic information, and harbour signalling molecules that target the behaviour of recipient cells [93,94,95]. Thus, they have recently emerged as critical paracrine conveyors of cell-to-cell information transfer in numerous biological systems. EVs can be isolated from in vitro cell-conditioned medium, as well as from plasma and several bodily fluids (e.g., urine, cerebrospinal fluid, saliva, and breast milk) [91]. EVs are mainly identified according to size which range from 35 nm to 1000 nm: very small (<200 nm) exosomes (Exo), medium-sized (200–500nm) MVs and larger sized apoptotic bodies (>500 nm) [96]. Within the Exo compartment, EVs can be further sorted into three subcomponents defined as large Exo (90–120nm, Exo-L), small Exo (60–80nm, Exo-S) and non-membranous nanoparticles called exomeres (about 35 nm) [94,97] that show distinct *N*-glycosylation, protein, lipid and genetic profiles.

Exos have a lipid bilayer membrane that envelops proteins and nucleic acid cargo, and originate within the endocytic pathway by originating from late endosomes. Endosomes that escape fusion with lysosome for degradation undergo a secondary membrane invagination leading to a bud of intraluminal vesicles forming the multi-vesicular body (MVB). MVB fuses its membrane with the cell membrane and releases Exo in the extracellular space. MVs are released by shedding from the plasma membrane by losing contact with the cytoskeleton. Apoptotic bodies resulting from the fractionation of the cellular content of cells undergoing programmed cell death. The specific driving mechanism for EV release is not yet completely understood [98]. It is markedly induced by various stimuli. For example, Ca^2+^ induces phospholipid redistribution and MV release in human erythrocyte membranes [99], while lipopolysaccharide (LPS) triggers MV release in monocytes [100]. In the vast majority of studies devoted to EVs, however, MVs and Exo were not discerned. This uncertainty reflects a lack of knowledge of the molecular mechanisms that drive the sorting of molecules into distinct EV populations. Despite the attention that has been dedicated to the analysis of the regenerative and/or pathological role of Exo among heterogeneous EVs, a consensus on the ideal strategy to isolate them as a pure preparation with optimal yield has not been reached yet. Therefore, in this review, we will more generally address heterogeneous Exo preparations as EVs when discussing the relevance of Exo-enriched populations.

Proteins transported by EVs include tetraspanins involved in MVB biogenesis, heat shock proteins and flotillins. Another important EV cargo comprises nucleic acid such as DNA (genomic and mitochondrial (mtDNA)) [100] and RNA, (including messenger RNA (mRNA), long noncoding RNA (lncRNA) and microRNA (miRNA)) [101,102]. Several of these molecules are common among all EVs (ExoCarta, http://www.exocarta.org) and are used as specific EV markers. Others are specific of the EV-producing cells and reflect their pathophysiological state. In addition, EVs released into the circulation and other bodily fluids display different RNA and protein contents in healthy subjects compared to patients with different diseases, which can be measured as potential biomarkers [103,104,105]. Notably, accumulating evidence points towards a key role of secreted EVs in mediating cell-communication processes, as well as in acting as indirect paracrine mediators of delivered cells within host tissue following cell therapy [91]. Thus, efforts to harness new “theranostic” applications have focussed on EVs as they combine accurate diagnostics with therapeutic effects [106]. 

### 2.2. Isolating Protocols and Their Impact on EV Biology

Effective methods for the isolation and characterisation of EVs remain challenging. To date, there is no consensus on standardised methods to separate the different vesicle sub-fractions in a reproducible way. As a result, isolation, characterisation and functional analysis of EVs have become a major focus in this research field [107,108]. 

The different methods for vesicle isolation take advantage of physical membrane properties of EVs. Ultracentrifugation, one of the most popular techniques used to isolate EVs from cultured cell-conditioned medium, is based on differential density [109,110,111]. Size exclusion chromatography (SEC) ultrafiltration separate EVs based on size [111,112]. Other methods are based on EV solubility when mixed to different substances, such as sodium acetate [113] or polyethylene glycol [114,115]. Immuno-capture beads have also been used for vesicle purification by targeting Exo surface markers [110]. Each isolation technique has its advantages and limits, which may impact EV biology and functional validation.

EV isolation via serial ultracentrifugation steps at increasing speeds (i.e., 10,000× *g* and 100,000× *g*) is the most commonly used technique, also for Exo enrichment [109]. Isolation by differential ultracentrifugation is also called the “pelleting method” or just the “ultracentrifugation method” [116] and it aims at separating medium-sized EV (which precipitate under 10.000× *g* acceleration) from nano-sized ones (which sediment at higher speed, 100,000× *g*). Ultracentrifuge can be performed with swinging or fixed-angle rotors. In order to pellet particles in a consistent and reproducible way, under different centrifugation conditions, the type of rotor should be set carefully, since rotor type and centrifugation time influences the yield and purity of extracellular vesicles [117]. At the end of the different ultracentrifugation steps, the EV pellet, which should be enriched with Exo, can be re-suspended in an appropriate solution, such as phosphate saline buffer (PBS), and stored at −80 °C or used immediately for further analyses. Variations of ultracentrifugation also exist, such as density gradient ultracentrifugation. A gradient can be created with sucrose or iodixanol. This latter improved the separation of EVs from other particles, such as apoptotic bodies, at all densities; hence, it may offer better preservation the vesicle size during their passage through the gradient [118]. In this method, samples are loaded on the top or on the bottom of a gradient in the centrifuge tube and upon applying centrifugal force, particles, including EVs, settle as individual zones through the density gradient. The separated vesicles can then be conveniently recovered by simple fraction collection. For example, EVs concentrate within a density gradient range of 1.10 and 1.21 g/mL gradient density [119]. After recovery from density gradient separation, the obtained EV fractions require further ultracentrifugation, according to the canonical pelleting method. Density gradient ultracentrifugation, as opposed to the canonical one alone, provides the cleanest EV samples that are suitable for detailed analyses, including omics technologies (from proteomics to RNA sequencing (RNAseq)). Classical ultracentrifugation may result in more contamination of proteins that can sediment along with EVs. Nevertheless, the more pelleting steps that are required, the higher the risk to compromise EV integrity for further investigation [120]. 

Stirred ultrafiltration is a simple and fast way to isolate EVs based on their size [121,122]. The pressure generated by the externally supplied nitrogen causes the sample to be passed through the ultrafiltration membrane resulting in EVs isolation. However, since the force applied may result in the deformation of vesicles, this could impact downstream analysis [123]. On the contrary, SEC is a gentler method allowing recovery of pure fractions. Samples are loaded on top of a sepharose solution and molecules smaller than the isolation range can be slowed down, as they enter into the pores of the stationary phase while larger particles, which are eluted from the column earlier [124]. SEC may be limited by the fact that EVs are recovered in a large collecting volume, thus further pelleting ultracentrifugation may be required to increase EV yield. EV isolation based on precipitation protocols is commonly available from commercial kits. This technique is definitely less time-consuming than serial ultracentrifugation or SEC, more user-friendly and does not involve specific laboratory equipment. While it is usually recommended for processing biological fluids, this method may be significantly affected by cross-contamination as a result of the precipitation technique itself. Immune-capture procedures have also been recently developed as addressing exosomal specific surface markers. Beads coated with specific antibodies are incubated with the biological samples and then pelleted in order to remove the unbound particles. Different types of beads are now available, such as magnetic beads [125], which allow simple removal of the unbound fraction, while increasing the probability of obtaining a cleaner EV sample. While being user-friendly and fast-acting, this method may be limited the following need of physical separation of captured EVs from the beads, thus affecting in vivo or in vitro analyses.

### 2.3. Unveiling the EV Cargo

**Figure 1:**
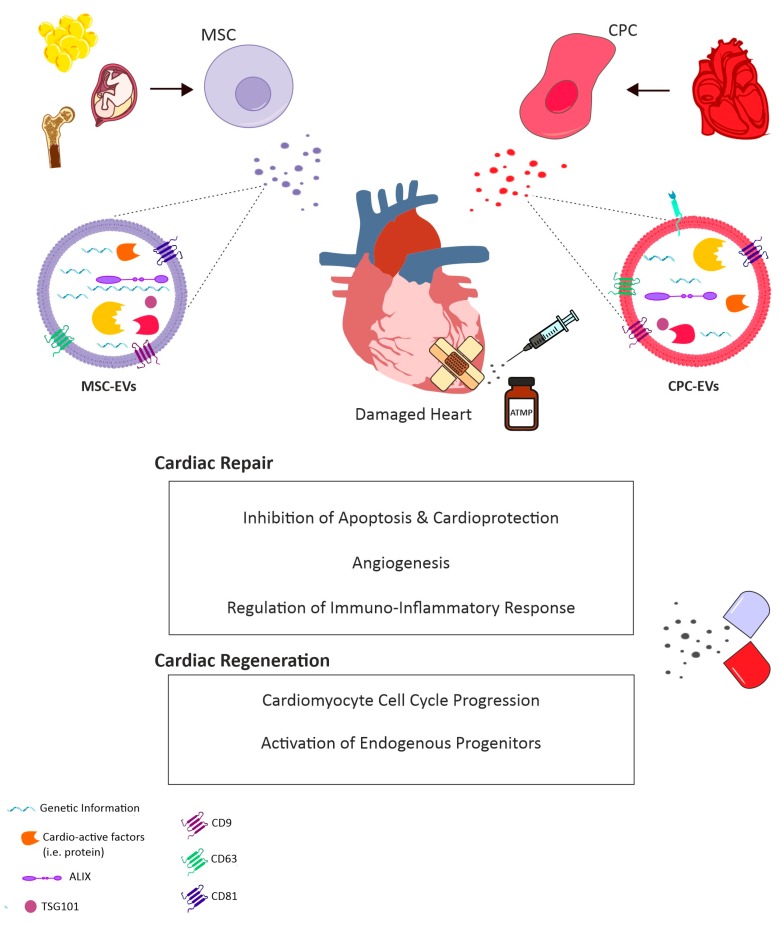
Schematic representation of the most relevant effects as part of cardiac repair mechanisms and cardiac regenerative effects delivered by administration into the injured heart of exogenous mesenchymal stromal cells (MSC)-extracellular vesicles (EVs) and cardiac progenitor cells (CPC)-EVs. ATMP: Advanced Therapy Medicinal Product.

As EVs represent very appealing theranostic tools, extensive effort has been made in characterising their biological content, especially under different conditions influencing their release from the parental cell, or when considering distinct secreting cells. EV protein cargo is influenced by their biogenesis pathway, thus protein involved in MVB formation are commonly found in Exo, such as Alix and TSG101, which can be then referred to bona fide EV/exosomal markers [126], along with tetraspanin CD63, CD81 and CD9 surface antigens (Figure 1) [96]. Results of proteomic analyses from several independent groups are included in comprehensive online databases such as EXOCarta (http://www.exocarta.org); vesciclepedia (http://www.microvesicles.org) and EVpedia (http://evpedia.info). EV protein content was at first investigated by gel electrophoresis separation and mass spectrometry [127,128,129,130]. Using these approaches, only 10–30 proteins per study were discovered in addition to the classical EV markers. In the last 10 years, protein separation and mass spectrometry technology have dramatically improved and the number of proteins found in EV samples has drastically increased [131]. Since EVs are only a small fraction of the entire cell secretome, contaminating soluble proteins may affect proteomic analyses. Contamination can occur from the original biofluids, as well as from the parental cell-conditioned medium. Providing that the number and type of the analysed protein are largely dependent on the type of isolation method used, technical standardisation, especially for sensible analyses such as proteomics, is urgently needed (extensively reviewed by Witwer et al. 2013) [132]. Another current major limitation for proteomic characterisation of vesicle content is represented by the lack of knowledge on the sorting mechanisms of specific cytosolic constituent into EV as messenger cargo. Nevertheless, different studies confirmed the presence of heat shock family proteins on EV surface [133]. HSP70, found on plasma EVs, can bind TLR4 on cardiomyocyte surfaces leading to activation of cardioprotective HSP27 [134]. Vicenio et al. identified the HSP70/TLR4 axis as a critical component of EV-mediated cardioprotection [134]. Moreover, HSP20 overexpressed on EVs derived from primary cardiomyocytes were promoted their proliferation, and migration and tube formation in endothelial cells [135]. Cardiac contractile dysfunction, observed in a streptozotocin-induced diabetes preclinical model, was significantly attenuated when transgenic mice overexpressed exosomal HSP20, compared to wild-type ones [135]. 

Genetic information is an important constituent of EV cargo, as delivered to the recipient cell via horizontal transfer. EVs are enriched with several RNAs that not always reflect the originating cell RNA profile [95,136,137,138], suggesting putative selectively incorporation into EVs. Yet, independent studies failed to demonstrate whether identified extracellular RNAs could be undeniably associated with EVs or rather with RNA–protein complexes which may have been co-isolated with EVs. Hence, RNAse treatment of EV pellet may be recommended [139]. Furthermore, since RNAseq on single EV may be technically challenging, the analyses performed so far can provide information on the average of the copy number obtained from a large number of heterogeneous EVs. This is particularly relevant in EVs obtained from biofluids, as these are released from different donor tissues and cells that can be contaminated by different lipoproteins associated with miRNAs [140]. As described earlier, different isolation methods can lead to extensive variation in the RNA yield. Lasser et al., in 2017, finely characterised extracellular RNA of pelleted EVs via classical ultracentrifugation and density gradient loading [141]. From the 10 fractions obtained, the detected RNA was divided into high density (HD) and low density (LD). Both fractions contained mRNA and miRNA, but mRNAs in the LD fraction correlated closely with the cellular mRNA, whereas the HD mRNA did not. Several other studies have extensively classified all the types of RNA present in isolated EVs [104,142]. 

Another relevant aspect concerning EV cargo is related to their putative mitochondrial content, including mtDNA. Mitochondria are vital for cell energy production by means of oxidative phosphorylation (OXPHOS). OXPHOS complexes on the invaginations of the mitochondrial inner membrane contain mitochondrial genome-encoded subunits [143,144,145,146]. It is known that compromised mitochondria can be eliminated by the autophagy/lysosome system in many cell types, including cardiomyocytes, in order to provide tissue protection [147,148]. Indeed, a highly selective type of autophagy, named mitophagy, can be activated following damage that targets the respiratory chain, alterations in membrane permeability and Ca^2+^ homeostasis, as well as mtDNA mutation [149]. Therefore, transfer of mitochondrial content via EVs may be specifically relevant as a paracrine pro-survival strategy within inter-cellular communication. Notably, EVs isolated from human mesenchymal stromal cells (MSCs) have been shown to carry mitochondrial functional respiratory complexes I, IV and V, suggesting their role in providing ATP synthesis restoration in compromised cells [150]. Moreover, MVs (i.e., >100nm in size) from human bone marrow MSC-conditioned medium were found enriched with mitochondria-like structures during mitophagy; likewise, human bone marrow MSC-EVs have been proven to enhance macrophage bioenergetics [151]. 

## 3. EVs for the Treatment of Cardiovascular Disease

### 3.1. EVs as Cell-Free Agents for Future Therapy

Therapeutic translation of EV biology into the clinical cardiovascular field has great potential for future therapy. Indeed, EVs can offer exclusive advantages, such as ensuring cell-specific paracrine effects on recipient cardiovascular cells, while overcoming most concerns and limits related to safety and feasibility of canonical cell transplantation, such as cell engraftment, survival and immunocompatibility. 

Currently, prognosis of heart failure following cardiac disease caused by MI remains poor, with dramatic costs for the national health system and with mortality estimated for 40% of patients within four years of diagnosis [152]. Indeed, a cardiovascular patient may need rapid intervention when facing MI via percutaneous coronary intervention, in order to limit cardiomyocyte loss. Prompt administration of cardio-active paracrine factors in the acute setting can significantly impact on early myocardial injury response, by preserving more viable tissue and sustaining local angiogenesis, thus limiting exacerbation of inflammation into chronic activation of myofibroblasts and spreading of fibrosis and scarring. This may result in effective cardioprotection while opposing detrimental remodelling, thus enhancing defective cardiac repair and inhibiting or at least delaying the onset of heart failure. As well, patients affected by non-ischaemic cardiomyopathy and/or cardiac dysfunction due to oncological treatment-derived cardiotoxicity may benefit from paracrine therapy to counteract premature senescence of resident cardiomyocytes and myocardial mitochondria impairment from increased oxidative stress.

Likewise, congenital heart defects, including very severe conditions (i.e., hypoplastic left heart syndrome) or those with relatively limited structural alterations (i.e., septal defects), may often result in paediatric heart failure. For defects requiring early treatment, the standard therapy is elective surgery within the first weeks of life using prosthetic implants to provide structural reconstruction [153]. However, such implants do not grow with the patient and additional surgeries are necessary with age. Thus, it would be of great advantage to support structural and functional myocardial reconstitution in these patients by stimulating resident cardiomyocyte renewal potential via paracrine stimulation.

In this scenario, EVs may represent a promising “off-the-shelf” next-generation advanced medicinal therapy product, which may be produced via a scale-up system from in vitro cultured cells to be promptly available as a pharmaceutical formulation for simple administration to the cardiovascular patient, when needed. Hence, the big question in preclinical research is focussing on identifying the most suitable stem/progenitor cell population to isolate EVs endowed with the most cardio-active paracrine potential. Here, we will review two main sources, endogenous CPCs versus exogenous stem/progenitor cells and discuss their capacity to impact cardiac intrinsic reparative and rejuvenating mechanisms via EV modulatory activity, as illustrated by the schematic in Figure 1.

### 3.2. A Role for CPC-Derived EVs

EVs released by primary cardiac stromal cells, obtained from atrial appendage and grown as monolayer [89] or by cardiosphere-derived cells (CDCs) [154], demonstrated reproduction of the cardioprotective and angiogenic effects of their parent cells both in vitro and in vivo. The benefits of CPC secretome in protecting cardiomyocytes from apoptosis have been attributed to their EV fraction acting through mechanisms involving both the transfer of nucleic acids and protein-protein interactions. For example, CPC-EVs are enriched in anti-apoptotic miRNA210, which acts by downregulating specific target genes in recipient cells such as ephrin A3 and PTP1b [89]. Likewise, miRNA132 in CPC-EVs was shown to exert a pro-angiogenic effect on endothelial cells by targeting RasGAP-p120 [89]. The surface protein defined as pregnancy-associated plasma protein-A (PAPP-A) was found to be associated within CPC-EV as master regulator responsible for the release of active IGF factor leading to activation of pro-survival pathways involving phosphorylation of ERK1/2 and Akt in recipient cells [155]. In a preclinical rat model of acute myocardial infarction (MI), CPC-EVs injected into the infarct border zone or systemically injected via tail vein increased viable mass and vessel density, resulted in a diminished scar and improved global heart function [154,156]. Ibrahim et al. showed that CDC-EVs were enriched in miRNA146a-5p. This miRNA was responsible for the increased viability of cardiomyocytes exposed to oxidative stress when treated with CDC-EVs as compared to controls. miRNA146a-5p downregulates specific genes in the target cells, including Traf6 and Irak1, two signalling mediators of the TLR-NFKB axis. Moreover, miR-146a knock-out mice showed larger infarcts compared to wild-type mice; injection of a miR-146a mimic at the time of MI rescued the level of ejection fraction similarly to wild-type animals, suggesting a mechanism of action for the cardioprotective effect mediated by CDC-EVs [90]. A similar effect has also been reported in a preclinical model of cancer drug-induced cardiotoxicity [157]. Indeed, human CPC-EVs were able to significantly decrease reactive oxygen species (ROS) production in cardiomyocytes following Doxorubicin/trastuzumab treatment in vitro. CPC-EVs prevented Doxorubicin-derived induction of Traf6, Smad4, Irak1, Nox4 and Mpo gene expression; this effect was abolished when miRNA146a-5p was knocked down via loading of a specific siRNA into the EV-producing cells. Moreover, in vivo intravenous administration of EVs significantly preserved cardiac function during Doxorubicin/trastuzumab treatment [157]. Additional studies have also identified Y RNA fragment inside CDC-EVs [158]. Such specific RNA was found to be actively transferred from EVs to macrophages and was able to induce macrophage polarisation into an M2 resolving phenotype. Y RNA provided cardioprotection by enhancing phagocytosis and scavenging debris from dying cells, thus attenuating damage from post-myocardial infarction [158]. CDC-EVs have been also tested in a porcine preclinical model, as delivered by intracoronary (i.c.) or open-chest intramyocardial (i.m.) routes 30 min after reperfusion, with the first being shown to be ineffective over the latter, in which EVs concurred to decrease infarct size and preserve left ventricular ejection fraction [159].

Priming of secreting CPCs via specific stimuli has also been suggested to improve EV biological activity; EVs produced by CPCs cultured for 12 h under hypoxia conditions were able to increase tube formation in endothelial cells and decrease pro-fibrotic gene expression in TGF-α stimulated fibroblast in vitro [160]. Microarray analysis identified eleven miRNAs upregulated in hypoxic preconditioned CPC-EVs compared to naive ones. Administration of hypoxic EVs into a preclinical model of ischaemia reperfusion injury (I/R) resulted in an improved cardiac functionality outcome compared to normoxic EVs [160]. Likewise, the potency of neonatal, infant or child human CPC-EVs under normoxic and hypoxic conditions has been also investigated. All three different population of EVs derived from CPCs exposed to hypoxia were more effective as compared to their normoxic counterpart, in terms of increasing new vessels formation and decreasing of fibrosis in a preclinical rat model of myocardial ischaemia/reperfusion injury (I/R) [161] With regards to their beneficial effects on cardiac function, neonatal CPC-EVs showed similar results in hypoxic or normoxic conditions [161]. While the cardioprotective effect of CPC-EVs has been broadly reported, very little is currently known about their putative effect on cardiomyocyte renewal. Positive effects of EVs on in vitro cultured rat neonatal cardiomyocytes have been described [90], together with encouraging data showing Ki67+ cardiomyocytes in an MI mouse model injected with CPC-EVs; yet the mechanism of action underlying such effect still needs clarification [162,163].

### 3.3. Contribution of Exogenous Stem/Progenitor Cell-EVs

CPCs have lately gained increasing attention as an appealing source for cardiac repair as being tissue-specific resident progenitors with significant paracrine activity, thus intrinsically likely to produce a more effective cardio-active secretome compared to other (somatic) sources. Despite the increasing number of studies supporting their EV cardiovascular modulatory potential, it is still a matter of debate whether they can represent the most suitable cell reservoir for future paracrine therapy. Indeed, isolation feasibility and self-renewal potential are key aspects of the ideal stem/progenitor secreting cell for clinical translation. In this perspective, as compared to other non-cardiac specific stem/progenitor cell sources, CPCs may be limited in their isolation from cardiac specimen via invasive surgical procedures or from cadaver donor supply and their limited proliferative potential. On the other hand, exogenous stem cells may offer an easily accessible and exploitable resource. 

In such scenarios, several populations have been investigated in the last years. Embryonic stem cell (ESC)-EVs have been described as positively modulating the cardiac healing process in a preclinical acute MI murine model. Notably, murine ESC-EVs not only supported local neovascularization, promoted cardiomyocyte survival and reduced scarring, but also enhanced CPC reactivation and their contribution to cardiac repair putatively via miR-294 delivery [164]; likewise, rodent ESC-EVs have been tested as an anti-inflammatory treatment in a preclinical mouse model of Doxorubicin-derived cardiomyopathy as quenching inflammasome protein expression leading to cardiomyocyte pyroptosis, as well as promoting the skewing of macrophages from pro-inflammatory M1 into pro-resolving M2 phenotype [165]. Since the discovery of induced pluripotent stem (iPS) cells in 2006 [166], they have been increasingly been tested as therapeutic agents for cardiovascular disease since overcoming major ESC concerns in terms of availability and ethical issues. More recently, on top of their plasticity, the paracrine potential of their secreted EVs has also been evaluated; indeed, mouse iPS cell-EVs have shown to exert cardioprotective effects on target cardiomyocyte both in vitro and in vivo by exosomal transfer of miR-21 and miR-210 as regulated by Nanog and HIF-1α [167]. Another strategy that has been lately pursued focusses on iPS cells as an exploitable “biopharmaceutical” source of (cardiovascular) progenitor cells and cardiomyocytes [168]. Indeed, multipotent CPCs have been generated from human iPS cells via stimulation of the small antioxidant molecule ISX-9; extracellular vesicles secreted by ISX-9-induced CPCs (EV-CPC^ISX-9^) counteracted cardiac fibrosis and ameliorated local angiogenesis in mice undergoing MI putatively via antifibrotic miR-373 delivery [169]. Similarly, iPS cell-derived cardiomyocytes (iCMs) have been broadly described as an alternative option for cell therapy approach [170]; since rodent cardiomyocytes have shown to secrete EVs biologically active on cardiovascular cells, iCM-EVs may offer an abundant paracrine source as well. Notably, a tissue engineering approach based on the application of an engineered hydrogel patch for the controlled release of iCM-EVs in a rat preclinical model of MI reduced infarct size while supporting cardiac function [171]. Despite such appealing results, EVs from iPS-derived cells may require extensive manipulation and can be cost-effective. 

Therefore, several somatic stem and tissue progenitor cells have been broadly investigated as a feasible alternative as EV biosource. Several preclinical and clinical studies have described beneficial modulatory effects of human bone marrow-derived CD34+ haematopoietic stem cells for cardiovascular disease, such as limiting angina frequency [172,173,174]. The therapeutic pro-angiogenic paracrine activity of human CD34+ stem cells in counteracting ischaemic injury has been shown to be recapitulated by their secreted EVs [175,176]. Given the relevance of therapeutic angiogenesis for de novo vessel formation following ischaemic injury, much interest has also been dedicated to the characterisation of endothelial progenitor cell (EPC)-EVs. Indeed, stimulation of cardiomyocytes undergoing Angiotensin II-induced hypertrophy and apoptosis with EPC-derived microvesicles resulted in improved survival as mediated by RNA-driven modulation of PI3K/Akt/eNOS pathway [177]. More recently, EPC-EVs have been administered to a rat model of MI within a shear-thinning hydrogel for their precise delivery and steady release, thus sustaining cardiac function and enhancing vessel density [178]. Notably, interleukin-10 deficiency within EPC-EVs has been recently shown to significantly affect their therapeutic influence for myocardial repair as altering their protein cargo and upregulating integrin-linked kinase-mediated activation of NF-κB in recipient cells; thus, modulation of the inflammatory priming of secreting EPC can impact on their EV profile and cardiovascular paracrine potential [179].

Haematopoietic stem and endothelial progenitor cells may represent promising cell sources; yet, their isolation yield might be limited by the need for specific stimulation. Hence, specific attention has been lately alternatively focussed on mesenchymal stromal cells (MSCs) obtained from either bone marrow (BM) or adipose tissue (AD) as easily reachable cell references for EV isolation. In particular, MSC-EVs have been shown to exert comparable, or even superior, therapeutic activities over parental MSCs in terms of quenching pro-inflammatory processes, oxidative damage and spread of fibrosis within the injured tissue [180]. With respect to ischaemic cardiovascular disease, MSC-EV treatment resulted in reduced myocardial apoptosis, and consequently decreased infarct size, with improved functional recovery and formation of new vessels, as extensively reviewed in [181]. Indeed, intra-myocardial delivery of EVs obtained from BM-MSCs has been reported to significantly modulate the cardiac microenvironment after acute myocardial infarction (MI), by stimulating neovascularisation and restraining the inflammation response [182]. Another relevant source of somatic MSCs is represented by the adipose tissue, since cells can be easily harvested by minimally invasive surgical techniques during lipoaspirate procedures. EVs obtained from AD-MSCs have been demonstrated protection of the myocardium against I/R injury via the Wnt/b-catenin signalling pathway [183]. As a matter of fact, the remarkably strong immunosuppressive effect of both BM- and AD-MSC-EVs on T cell activation and macrophage polarisation has been documented as one of their major beneficial effects [184,185,186]. Increasing evidence has indicated that the EV microRNA (miRNA) content as one of their most likely paracrine mechanism(s) of action. Several miRNAs have been associated with MSC-EV cardioprotective, pro-survival and angiogenic effects, including, for example, miR-21 [187]; miR-125b [188]; miR-320d [189]; miR-95–3p [190]; miR-210 [191]; miR-29 and miR-24 [180]. Additionally, EVs have been shown to biochemically restore ATP and NADH levels and redox state within the injured tissue [192]. 

Given the remarkable cardio-active effects of EVs, different methods have been lately explored to boost their therapeutic exploitation by ad hoc preconditioning or engineering of parental MSCs. Indeed, somatic MSCs have been stimulated either under hypoxic and/or pro-inflammatory conditions, resulting in the implementation of their secreted EVs to provide tissue repair, along with modulatory and pro-angiogenic effects [188,193,194,195]. Pharmacological priming by atorvastatin, a well-established lipid-lowering drug that can enhance stromal progenitor cell cardioprotective effects, has also been reported [196]. Another well-accepted approach is to increase EV function via genetic overexpression of specific miRNAs within parental MSC as vesicle cargo loading strategy; as result, a significant fold enrichment of those regulatory miRNAs putatively involved in EV paracrine ability has been observed, thus enhancing their phenotypic effects on responder cells [189,190,197].

Adult somatic or embryonic stem cells may be limited by low yield, invasive sampling, controversial self-renewal and ethical issues. Stromal progenitors isolated from foetal and perinatal extra-embryonic tissues can offer an ideal alternative as a leftover sample from prenatal screening procedures during gestation or as waste material after birth. Indeed, foetal and perinatal MSCs are immature progenitors with high self-renewal potential, have significant immunomodulatory properties and distinct pro-angiogenic, cytoprotective and anti-inflammatory paracrine profile. Foetal and perinatal sources include amniotic fluid and term umbilical cord and placenta, respectively, from which MSC can be easily isolated without any ethical concern [198]. EVs from umbilical cord-derived MSCs (UC-MSC-EVs) have been recently broadly scrutinised in terms of their beneficial effects in different preclinical animal models of myocardial injury. Human UC-MSC-EVs overexpressing the pro-survival Akt kinase was shown to modulate local angiogenesis in a preclinical rat model of MI, via PDGF-D as pro-angiogenic mediator [199]. Likewise, EVs from UC-MSCs overexpressing the tissue matrix metalloproteinase inhibitor 2 (TIMP2) or the cardioprotective stromal-derived factor 1 alpha (SFD1a) have been shown to limit detrimental ventricular remodelling via the pro-survival Akt/Sfrp2 pathway and to inhibit apoptosis and autophagy of myocardial cells while sustaining local angiogenesis in preclinical rodent models of MI [200,201]. MSCs derived from human term placenta, also referred to as amniotic mesenchymal stromal cells (AMSCs), are well-known for their (immuno)modulatory properties [202,203]; EVs released by human term placenta-MSCs have been recently demonstrated exertion of relevant therapeutic effects as supporting new vessel development in vitro and in vivo [204], with nitric oxide (NO)-releasing polymer stimulation as a functional trigger of exosomal enrichment of pro-angiogenic VEGF and miR-126 [205]. Notably, placenta-MSCs and their derived EVs have been shown to counteract skewing of myoblasts to fibrogenic phenotype and collagen IV expression in the cardiac tissue of preclinical models of Duchenne musclar dystrophy, via targeted delivery of miR-29c [206].

Notably broadly multipotent MSCs with remarkable proliferative capacity along with pro-angiogenic, anti-inflammatory and cardioprotective modulatory potential have been described as isolated from human amniotic fluid, namely c-KIT+ human amniotic fluid-derived stem cells (hAFSCs) [207,208,209,210,211,212,213]. hAFSCs can be easily isolated from leftover samples of amniotic fluid obtained from prenatal screening amniocentesis (i.e., foetal hAFSCs) or from clinical waste during eligible caesarean delivery without any ethical concern (i.e., perinatal hAFSCs). In particular, hAFSC-EVs have been reported to enhance cell survival in preclinical models of kidney, lung and skeletal and cardiac muscle injury, possibly via the horizontal transfer of their miRNA cargo, including cardio-active miR-199a-3p and miR-210 [83,214,215,216]. In mice undergoing MI, single intramyocardial administration of hAFSC-EVs obtained following cell preconditioning under hypoxia resulted in prolonged beneficial effects ameliorating cardiac repair mechanisms and inhibiting worsening of myocardial function in the long term [83]; hence, being hAFSCs immature and “developmentally young” progenitors, they may possess a powerful paracrine potential for myocardial “rejuvenation” strategies and represent an appealing cell source for future therapy.

Notably, fewer studies have specifically addressed the MSC-EV regenerative potential to locally reactivate in vivo endogenous CPCs, along with stimulation of resident cardiomyocyte renewal. Human miR-590–3p has been previously reported to impact myocardial reconstitution by downregulating genes inhibiting cardiomyocyte proliferation; indeed, based on their miR-590–3p enrichment, EVs obtained from MSC genetically engineered to express a cardiac troponin-targeting peptide showed to target cardiomyocyte cell cycle re-entry and sustained cardiac function in a preclinical MI model [217]; similarly, miR-199a-loaded BM-MSC-EVs have demonstrated support of rodent cardiomyocyte cell cycle progression in vitro via dose-dependent manner by means of Crim1 and Caspase-9 downregulation [197]). Likewise, hAFSC-EVs were able to stimulate endogenous regenerative mechanisms from within the injured murine myocardium by reactivating epicardial WT1+ CPCs paracrine activity and triggering resident cardiomyocyte cell cycle progression up to DNA duplication phase, both at early and longer time points after MI [83]. Since structural and functional cardiomyocyte reconstitution is the sine qua non condition for true cardiac regeneration, further investigations are required to carefully define the EV potential in sustaining bona fide cardiomyocyte duplication by rigorous evaluation of complete cytokinesis over defective karyokinesis with DNA duplication resulting in binucleation/polyploidy.

### 3.4. Looking for the Right Address: Improving EV Cardiac Tropism 

A critical aspect to be considered in clinical translation of EVs for future cardiovascular medicine is represented by their quite limited homing potential when delivered systemically. 

Intra-myocardial administration of EVs soon after injury (i.e., myocardial infarction) has been shown to provide beneficial paracrine effects addressing both cardiac repair and regenerative mechanisms, resulting in long-term effects after a single acute injection [89,90,155,218]. Nevertheless, paracrine effects are well known to act promptly and locally, therefore follow-up administration would be the ideal treatment regime, especially to modulate cardiac detrimental pathological cardiac remodelling over time. Several ideas have been put forward to overcome the low EV retention rate after transplantation in vivo by suggesting, for example, functional vesicle encapsulation in optimised hydrogel formulation to ensure topical sustained release over time [219]. It is important to bear in mind that not all cardiovascular patients may be eligible for acute in situ treatment requiring either percutaneous coronary intervention/angioplasty or collateral cardiac surgery; therefore, there is an increasing need for optimizing EV cardiac-specific homing for future cell-free paracrine therapy. Several studies have been lately reported suggesting alternative strategies to functionalise EVs for more accurate targeting of the damaged heart [220,221]. These include: lentiviral vector-based engineering of secreting cells to upregulate the expression of cardiomyocyte-specific binding peptides fused to the murine transmembrane protein Lamp2b, in order to enrich the targeting epitope on the exosomal surface [222]; overexpression of exosomal CXCR4 to push their bioavailability towards the ischaemic heart [156]; membrane anchoring systems to directly dock tissue-specific antibodies or homing antigens on the EV surface [223,224]. 

Notably, a very elegant work has recently translated CAR-T cell technology beyond oncology into the cardiovascular field, to specifically address and treat cardiac fibrosis via the engineering of CAR receptor against a cardiac endogenous protein activating resident fibroblasts [225]. Likewise, CAR-EVs, as derived from CAR-T cells, have also been reported as maintaining target specificity with a much lower risk of cytotoxic cytokine release syndrome commonly caused by adoptive CAR-T cell therapy [226]. This may shed new light on the development of novel strategies to enhance EV cardiac tropism as putative future advanced medicinal paracrine therapy products to counteract cardiovascular disease and heart failure.

## 4. Conclusions: Challenges to be Overcome

The analytical study of EVs is a very active area of research. Functional readouts of biomedical applications of EV obtained by either CPCs or exogenous stromal cells of non-cardiac origin have revealed that both types may harbour therapeutic relevance for cardiovascular disease, questioning the rationale that CPCs could have superior cardio-active effects. Yet, some additional aspects are in need of further clarification in order to achieve a general consensus. Indeed, in order to develop an EV-based therapeutic approach, a comprehensive characterisation of the tissue/cellular source of EVs is imperative. Detailed methods to obtain human cells from different tissues origin are well reported; however, donor-to-donor variability remains a prominent challenge. BM-MSCs from aged mice have demonstrated reduced wound healing, angiogenesis, proliferation and anti-apoptotic capabilities [227]; similarly, human adipose tissue MSCs, derived from patients at different ages, showed remarkably reduced in vitro differentiation potential [227]. Therefore, age and degree of developmental/maturation commitment can affect stem and progenitor cell reparative and regenerative potential; how this specifically impacts the therapeutic capacity of derived EVs remains to be specifically defined. Currently, there is little knowledge of how co-purified non-EV associated molecules affect the activity of the samples. Furthermore, it is reasonable to think that only a portion of EVs mediate the expected therapeutic effect while others could act in an antagonistic manner. Indeed, due to a current lack of practical technologies to analyse EVs at the single vesicle level, the heterogeneity of EV fractions cannot be comprehensively addressed, even if they are harvested from apparently homogeneous cell sources. Proper in vitro and in vivo tests are required to predict the intended therapeutic potential, along with safety and potency, of the EV fractions. Moreover, one of the most relevant therapeutic advantage of EVs, compared to the parental cells, is their potential to “escape” to the immune system. The absence of costimulatory molecule MHC-II gives EVs the potential to escape recognition by CD4 lymphocytes [90,216,228], although this aspect needs more detailed investigation.

A key point to be considered is the current lack of standardised procedure available for EV isolation, as well as for their storage. For instance, there is evidence that independent EVs preparation shows different immunomodulatory capability [229]. The International Society of Extracellular Vesicles (ISEV), in 2017, in a position paper [88], addressed some aspects of safety and regulatory requirements that must be considered for clinical application. EV-based therapeutics are now subjected to regulatory frameworks concerning biological medicinal products; however, there is a consensus that special guidelines for EV-based therapeutics may be needed. Moreover, the clinical translation of EVs requires standardisation of administration and dosing. Nowadays, for liposome-encapsulated forms of doxorubicin [230], methods such as TRPS are currently used to verify particle size and characterisation and are accepted by FDA and EMA, yet for EVs, regulatory guidance is lacking.

Eventually, the development of a good manufacturing practice (GMP)-grade method for the large-scale preparation of EVs as an advanced therapy medicinal product (ATMP, Figure 1) is also mandatory to implement their bench-to-bedside application. The first evidence of this has been recently performed for CPC-EVs. Indeed, human CPCs cultured in xeno-free conditions showed production of EVs with similar features as in research-grade conditions [228]. The GMP method guaranteed high exosome yield (>1013 particles) by isolating through a closed system of ultrafiltration, and consistent removal (≥97%) of contaminating proteins. Thus, such standardised production method for large-scale manufacturing of CPC-EVs may offer a good demonstration of the clinical translation of EV biology for human therapeutic applications for the treatment of acute myocardial infarction syndrome [228].
